# Estren promotes androgen phenotypes in primary lymphoid organs and submandibular glands

**DOI:** 10.1186/1471-2172-6-16

**Published:** 2005-07-12

**Authors:** Ulrika Islander, Bengt Hasséus, Malin C Erlandsson, Caroline Jochems, Sofia Movérare Skrtic, Marie Lindberg, Jan-Åke Gustafsson, Claes Ohlsson, Hans Carlsten

**Affiliations:** 1 Department of Rheumatology and Inflammation Research at the Sahlgrenska Academy, Göteborg University, Sweden; 2 Institute of Odontology, Sahlgrenska Academy, Göteborg University, Sweden; 3 Center for Bone Research at the Sahlgrenska Academy (CBS), Göteborg University, Sweden; 4 Center for Biotechnology and Department of Medical Nutrition, Karolinska Institute, NOVUM, Huddinge, Sweden

## Abstract

**Background:**

Estrogens and androgens have extensive effects on the immune system, for example they suppress both T and B lymphopoiesis in thymus and bone marrow. Submandibular glands are sexually dimorphic in rodents, resulting in larger granular convoluted tubules in males compared to females. The aim of the present experiments was to investigate the estrogenic and androgenic effects of 4-estren-3α,17β-diol (estren) on thymus, bone marrow and submandibular glands, and compare the effects to those of 17β-estradiol (E2) and 5α-dihydrotestosterone (DHT), respectively. Estrogen receptors (ERs) were blocked by treatment of mice with the ER-antagonist ICI 182,780; also, knock-out mice lacking one or both ERs were used.

**Results:**

As expected, the presence of functional ERs was mandatory for all the effects of E2. Similar to DHT-treatment, estren-treatment resulted in decreased thymus weight, as well as decreased frequency of bone marrow B cells. Treatment with estren or DHT also resulted in a shift in submandibular glands towards an androgen phenotype. All the effects of estren and DHT were independent of ERs.

**Conclusion:**

Our study is the first to show that estren has similar effects as the androgen DHT on lymphopoiesis in thymus and bone marrow, and on submandibular glands, and that these effects are independent of estrogen receptors. This supports the hypothesis of estren being able to signal through the androgen receptor.

## Background

The effects of estrogens and androgens on the immune system in mice have been extensively studied. For example, B and T lymphopoiesis in bone marrow and thymus is suppressed by treatment with both estrogens [1-6] and androgens [7-9].

Submandibular glands (SMG) are sexually dimorphic in rodents. The secretory activity of these glands is mainly localized to the acinar cells and the granular convoluted tubular (GCT) cells. The GCT cells are under hormonal control involving androgens, resulting in larger GCT in males compared to females [10-12].

Signals from estrogens and androgens are transmitted into the target cells by the two known estrogen receptors (ERs), ERα and ERβ [13,14], or by the androgen receptor (AR), respectively.

Postmenopausal hormone replacement therapy (HRT) has beneficial effects on the skeleton, but is associated with well-known side effects. This has lead to an increased focus on finding synthetic estrogen-like substances that only reproduce the beneficial effects of estrogen. 4-estren-3α,17β-diol (estren) is a synthetic compound with structural similarities to E2 that has been suggested to signal through both ERs and the AR [15-17].

The aim of the present experiments was to investigate the estrogenic and androgenic effects of estren on lymphopoiesis in thymus and bone marrow, and on SMG. We show here that estren has similar effects on these organs as the androgen DHT, and that the effects are independent of ERs, supporting the previous studies showing that estren is able to signal through the AR[15,16].

## Results

### Experiment 1: Estren does not affect thymus, bone marrow B cells or submandibular glands through ERs

We wanted to investigate the estrogenic effects of estren on lymphopoiesis and submandibular glands (SMG) in female mice. In order to do this, 3-month-old female C57/B16 mice were ovariectomized and treated during 18–21 days with daily subcutaneous (s.c.) injections of the estrogen receptor antagonist ICI 182,780 (200 μg/day) or vehicle Miglyol 812. Simultaneously, both the vehicle and the ICI groups were given daily s.c. injections of E2 (0.7 μg/day), estren (75 μg/day) or control Miglyol 812 oil.

**Figure 1:**
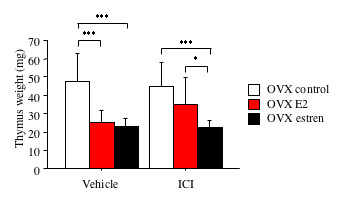
**Estren reduces thymus weight independently of estrogen receptors. **Ovariectomized 3-month-old female C57/B16 mice were treated for 18–21 days with daily s.c. injections of the estrogen receptor antagonist ICI 182,780 (200 μg/day) or vehicle Miglyol 812. Simultaneously, both the vehicle and the ICI groups were given daily s.c injections of either E2 (0.7 μg/day), estren (75 μg/day) or control Miglyol 812 oil. ICI 182,780 blocked the inhibitory effect of E2 on thymus weight. Treatment with estren resulted in lower thymus weight in both vehicle and ICI mice. One-way ANOVA followed by Fisher's test was used to compare data from mice in different treatment groups. Results are presented as mean ± standard deviation. * *P *< 0.05, *** *P *< 0.001.

**Figure 2:**
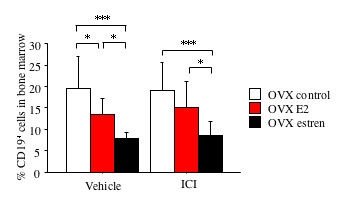
**Estren reduces the frequency of CD19^+ ^B cells in bone marrow independently of estrogen receptors. **Ovariectomized 3-month-old female C57/B16 mice were treated for 18–21 days with daily s.c. injections of the estrogen receptor antagonist ICI 182,780 (200 μg/day) or vehicle Miglyol 812. Simultaneously, both the vehicle and the ICI groups were given daily s.c injections of either E2 (0.7 μg/day), estren (75 μg/day) or control Miglyol 812 oil. ICI 182,780 blocked the inhibitory effect of E2 on the frequency of CD19^+ ^B cells in bone marrow. Treatment with estren resulted in lower frequency of CD19^+ ^B cells in bone marrow in both vehicle and ICI mice. One-way ANOVA followed by Fisher's test was used to compare data from mice in different treatment groups. Results are presented as mean ± standard deviation. * *P *< 0.05, *** *P *< 0.001.

The inhibitory effects of E2 on thymus weight (fig. 1) and frequency of CD19^+ ^cells in bone marrow (fig. 2) in vehicle-exposed mice were blocked by the ER-antagonist ICI 182,780. In contrast, treatment with estren resulted in lower thymus weight (fig. 1) and lower frequency of CD19^+ ^cells in bone marrow (fig. 2), in both vehicle and ICI exposed mice.

**Figure 3:**
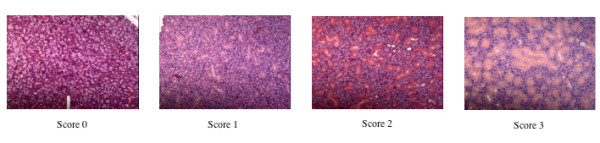
**Representative photos of the androgen phenotype scoring points. **The picture shows representative photos of the scorings (0–3) used to evaluate the degree of androgen phenotype in eosin-hematoxylin stained SMG sections. The degree of the androgen phenotype in the SMG sections were scored from 0 = no androgen phenotype, to 3 = maximal androgen phenotype.

**Figure 4:**
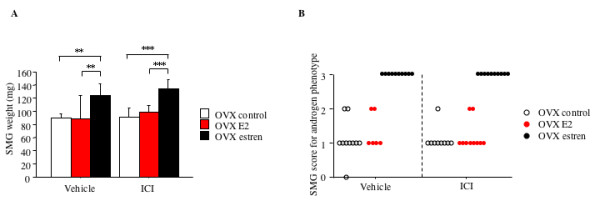
**Estren induces increased submandibular gland weight and androgen phenotype independently of estrogen receptors. **Ovariectomized 3-month-old female C57/B16 mice were treated for 18–21 days with daily s.c. injections of the estrogen receptor antagonist ICI 182,780 (200 μg/day) or vehicle Miglyol 812. Simultaneously, both the vehicle and the ICI groups were given daily s.c injections of either E2 (0.7 μg/day), estren (75 μg/day) or control Miglyol 812 oil. Treatment with E2 did not affect the SMG weight **(A) **or score for androgen phenotype **(B) **in either vehicle or ICI mice. Estren-treatment resulted in increased SMG weight **(A) **and SMG score for androgen phenotype **(B) **in both vehicle and ICI mice. One-way ANOVA followed by Fisher's test was used to compare data from mice in different treatment groups **(A)**. Results are presented as mean ± standard deviation. **P < 0.01, *** *P *< 0.001.

The granular convoluted tubules in SMG are sexually dimorphic in rodents, being larger in males than in females. In eosin-hematoxylin stained tissue sections of SMG, the degree of the androgen phenotype was scored from 0 to 3. 0 represents no androgen phenotype and 3 is the highest score for androgen phenotype. Representative pictures for the different scoring points are presented in fig. 3. Treatment with E2 did not affect the SMG weight (fig. 4a) or score for androgen phenotype (fig. 4b) in either vehicle or ICI mice. Furthermore, both the SMG weight (fig. 4a) and the SMG score for androgen phenotype (fig. 4b) was increased by estren in both vehicle and ICI mice.

Taken together, results from this experiment indicate that estren affects lymphopoiesis and SMG through ER-independent pathways.

### Experiment 2: Estren and DHT affect the frequency of B220^+ ^cells in bone marrow, and submandibular gland phenotypes, through ER independent pathways

Since previous studies have shown that estren has the ability to signal through both ERs and AR[15,16], we wanted to investigate the estrogen-, and androgen-like effects of estren in female ER knock-out mice. In order to do this, 11-month-old female WT and DERKO (ERα^-/-^, ERβ^-/-^) mice were ovariectomized and given daily s.c. injections of E2 (0.7 μg/day), DHT (120 μg/day) or estren (75 μg/day) during four weeks. Control mice received olive oil.

**Figure 5:**
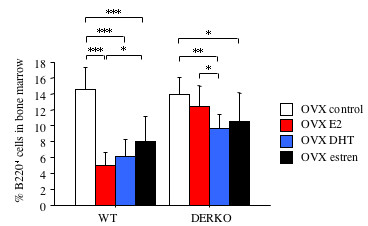
**DHT and estren reduce the frequency of B220^+ ^cells bone marrow independently of estrogen receptors. **Ovariectomized 11-month-old female WT and DERKO mice were given daily s.c injections of E2 (0.7 μg/day), DHT (120 μg/day) or estren (75 μg/day) during four weeks. Control mice received olive oil. As expected, the E2 mediated reduction of B220^+ ^cells in bone marrow of WT mice was lacking in DERKO mice. Treatment with DHT or estren resulted in a lower frequency of B220^+ ^cells in bone marrow of both WT and DERKO mice. One-way ANOVA followed by Fisher's test was used to compare data from mice in different treatment groups. Results are presented as mean ± standard deviation. * *P *< 0.05, * *P < 0.01, *** *P *< 0.001.

**Figure 6:**
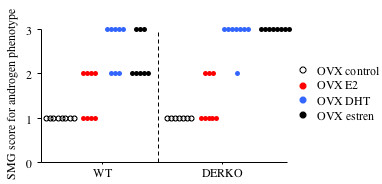
**DHT and estren increase the androgen phenotype score in submandibular glands independently of estrogen receptors**. Ovariectomized 11-months-old female WT and DERKO mice were given daily s.c. injections of E2 (0.7 μg/day), DHT (120 μg/day) or estren (75 μg/day) during four weeks. Control mice received olive oil. E2 treatment did not dramatically affect the androgen phenotype of SMG, in either WT or DERKO mice. THE SMG score for androgen phenotype was high for both DHT- and estren-treated mice, of both WT and DERKO genotype, when compared to control mice.

As expected, the E2-mediated reduction of the frequency of B220^+ ^cells in bone marrow of WT mice was lacking in DERKO mice (fig. 5). E2-treatment did not dramatically affect the weight (data not shown) or the androgen phenotype of SMG in either WT or DERKO mice (fig. 6).

We have previously shown that estren-treatment results in decreased thymus cellularity in both WT and DERKO mice indicating an effect through ER independent pathways[17]. We now also show that treatment with DHT or estren resulted in lower frequency of B220^+ ^cells in bone marrow of both WT and DERKO mice (fig. 5). Furthermore, fig. 6 shows that the SMG score for androgen phenotype was high for both DHT- and estren-treated mice, of both WT and DERKO genotype, when compared to control mice. Interestingly, the SMG weight was significantly increased in DHT-treated mice compared to controls, but not in estren- or E2-treated mice (data not shown).

Altogether, results from this experiment show again that estren affects B cells in bone marrow, and the phenotype of SMG, through ER-independent pathways in a manner similar to DHT-treatment. This indicates estren-signalling through the AR.

### Experiment 3: DHT treatment results in decreased thymus cellularity and an androgen phenotype of submandibular glands

In order to study how E2- and DHT-treatment affected male ER knock-out mice, 9-month-old male WT and DERKO mice were orchidectomized and treated for 4 weeks with E2 (0.05 μg/day) or DHT (45 μg/day), administered by s.c. silastic implants in the cervical region[18]. Vehicle animals received empty implants.

**Figure 7:**
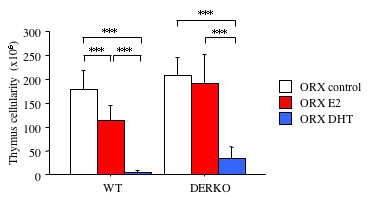
**DHT reduces thymus cellularity independently of estrogen receptors**. 9-month-old male WT and DERKO mice were orchidectomized and treated for 4 weeks with E2 (0.05 μg/day) or DHT (45 μg/day), administered by s.c. silastic implants. Vehicle animals received empty implants. The E2-mediated reduction of thymus cellularity seen in WT mice, could not be detected in DERKO mice. Treatment with DHT resulted in a dramatic reduction of thymus cellularity independently of ER genotype. One-way ANOVA followed by Fisher's test was used to compare data from mice in different treatment groups. Results are presented as mean ± standard deviation. ***P < 0.001

**Figure 8:**
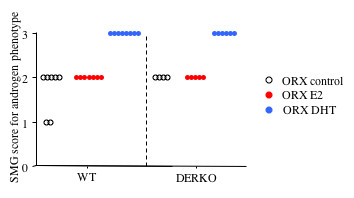
**DHT increases the score for androgen phenotype in submandibular glands independently of estrogen receptors. **9-month-old male WT and DERKO mice were orchidectomized and treated for 4 weeks with E2 (0.05 μg/day) or DHT (44 μg/day), administered by s.c. silastic implants. Vehicle animals received empty implants. E2-treatment did not affect the androgen phenotype of SMG in either WT or DERKO mice. The SMG score for androgen phenotype was increased in DHT treated mice of both WT and DERKO genotype.

The E2-mediated reduction of thymus cellularity seen in WT mice could not be detected in DERKO mice (fig. 7). However, E2-treatment did not affect the weight (data not shown) or androgen phenotype of SMG in either WT or DERKO mice (fig. 8).

Treatment with DHT resulted in dramatically reduced thymus cellularity independent of ER genotype (fig. 7). Furthermore, fig. 8 shows that the SMG score for androgen phenotype was increased in DHT-treated mice of both WT and DERKO genotype. The SMG weight was also significantly increased in DHT-treated mice of both ER genotypes compared to control- and E2-treated mice (data not shown).

In conclusion, these data show that also in male mice DHT affects both thymus cellularity, and SMG weight and phenotype, independently of ERs.

### Experiment 4: Aged ERKO mice have increased salivary gland weight that displays an androgen phenotype

Finally, untreated 18-month-old WT, ERKO, BERKO, and DERKO mice were investigated. We found that aged non-ovariectomized female mice lacking ERα (ERKO) displayed an increased SMG weight, size and an androgen histological phenotype (9a-c). We have previously shown that female ERKO mice have higher serum levels of testosterone compared to WT mice[19]. Therefore, these results indicate that high levels of testosterone in ERKO mice are responsible for the increased SMG weight, size and androgen phenotype in aged female ERKO mice.

## Discussion

Postmenopausal hormone replacement therapy (HRT) has beneficial effects on the skeleton but is associated with well-known side effects. This has lead to an increased focus on finding synthetic estrogen-like substances that only reproduce the beneficial effects of estrogen. Estren is a synthetic compound that we have recently shown to have ER-dependent suppressive effects on inflammation, but ER-independent inhibitory effects on T lymphopoiesis [17]. The aim of the present experiments was to investigate the estrogenic and androgenic effects of estren on lymphopoiesis in thymus and bone marrow, and on SMG. Our study is the first to show that estren has similar effects on these organs as the androgen DHT, supporting the hypothesis that estren is able to signal through the AR.

In genomic signalling pathways, estrogen binds to intracellular or – possibly - membrane bound ERs, ultimately activating transcriptional activity in the target cell. However, reports have shown that a variety of cell types respond rapidly, within seconds or minutes, making the genomic signalling pathway unlikely and suggesting instead non-genomic signalling pathways. In previously published papers, Kousteni *et al *[20-22] proposed that sex steroids affect reproductive tissues by classical genomic signalling, while the bone sparing effect of sex steroids is mediated through a non-genomic pathway. It was suggested that ERα, ERβ or AR can activate the non-genomic signalling pathway irrespective of whether the ligand is an estrogen or an androgen. They also showed that treatment with estren increases bone mass in ovariectomized mice without affecting reproductive organs, suggesting that estren is a "mechanism specific" compound that only reproduces the non-genomic signalling of estrogen, and thus can affect target cells also through the AR.

In constrast to the findings by Kousteni *et al*, we have previously shown that estren has the capacity to affect both bone and reproductive organs through classical genomic signalling via ERs[23].

Furthermore, Centrella *et al*[15] found that estren activates osteoblasts in a genomic and AR-dependent way. Interestingly, they also showed that estren can be metabolized into 19-nortestosterone by action of 3α-hydroxysteroid dehydrogenase, and that 19-nortestosterone binds to AR with an affinity that was approximately 40% of that of DHT.

In a recently published paper, Krishnan *et al*[16] found estren to be a potent AR ligand with the ability to translocate AR into the nucleus, and possess full AR agonist activity. In conclusion, results from these studies show that the ER-independent effects of estren may be mediated through the AR.

We have recently demonstrated that estren affects peripheral immune functions through ERs, while the inhibitory effects on T lymphopoiesis is ER-independent[17]. Since also AR stimulation is known to down-regulate both T and B cell development [7-9], the non ER-mediated effects of estren on thymus may results from activation of AR.

In the present report we have studied the estrogenic and androgenic effects of estren on thymus, B cells in bone marrow and on SMG. We show here that blocking of ERs with ICI 182,780 does bot affect the estren-induced inhibition of thymus weight (fig. 1). We have previously shown that exposure to estren results in decreased thymus cellularity independently of ERs[17]. In this study we also show that treatment with DHT results in decreased thymus cellularity in both WT and DERKO mice (fig. 7).

Similarly, we show here that blocking of ERs with ICI 182,780 does not affect the frequency of CD19^+ ^B cells in bone marrow (fig. 2), and independently of ERs, both DHT- and estren-treatment results in lower frequency of B220^+ ^B cells in bone marrow (fig. 5).

SMG are sexually dimorphic in rodents, resulting in larger granular convoluted tubules in males compared to females [10-12]. In this study, the SMG sections were scored according to their androgen phenotype (fig. 3) and the results show that blocking of ERs with ICI 182,780, does not affect the increased score of androgen phenotype (fig. 4b) or increased SMG weight (fig. 4a) seen after exposure to estren. We also show that treatment with estren or DHT of both ovariectomized female and orchidectomized male mice, results in an androgen phenotype (fig. 6 and 8) in both WT and DERKO mice. Interestingly in experiment 2, estren-treated WT and DERKO mice did not display increased SMG weight in correlation to their increased androgen phenotype. The reason for this is not clear, but one possible explanation could be that a longer treatment period is necessary to induce the increase in SMG weight of aged female mice.

**Figure 9:**
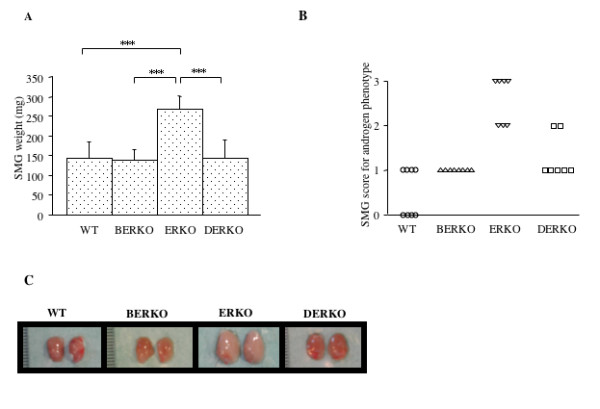
**Untreated aged female mice lacking ERα display androgen phenotype of submandibular glands. **Untreated 18-month-old WT, ERKO, BERKO, and DERKO mice were used in the experiment. Aged female mice lacking ERα (ERKO) displayed an increased SMG weight **(A)**, size **(C) **and androgen phenotype **(B)**. One-way ANOVA followed by Fisher's test was used to compare data from mice of different genotypes in **(A). **Results are presented as mean ± standard deviation. *** *P *< 0.001

In experiment 4, we demonstrate that aged untreated female mice lacking ERα have an androgen SMG phenotype (fig. 9a–c). This is very likely due to the increased testosterone levels found in these mice [19].

## Conclusion

We have recently demonstrated that estren affects peripheral immune functions through ERs, while the inhibitory effects on T lymphopoiesis is ER-independent[17]. Since AR-stimulation is known to down-regulate both T and B cell development [7-9], and it has been shown that estren has the ability to signal also through the AR[15,16], the non ER-mediated effects of estren may result from activation of AR. In this study we show that estren has similar non ER-mediated effects on lymphopoiesis as the androgen DHT in both thymus and bone marrow, and on the phenotype of SMG. This strongly supports the hypothesis of estren having the capacity to signal through the AR.

## Methods

The ethical committee for animal experiments at Göteborg University approved this study. Mice were kept in the animal facility at Göteborg University under standard conditions of temperature and light, and had free access to fresh water and standard laboratory chow or soy-free food pellets R70 (Lactamin AB, Stockholm, Sweden).

### Mice and treatments

#### Experiment 1

3-month-old female C57/B16 mice were ovariectomized under Ketalar^® ^(Pfizer AB, Täby, Sweden) /Domitor^® ^(Orion Pharma, Espoo, Finland) anaesthesia and left to rest for two weeks before start of the experiment. Mice were given daily s.c. injections of the ER-antagonist ICI 182,780 (Tocris Cookson Ltd., Bristol, UK) (200 μg/day) or vehicle Miglyol 812 (generous gift from Recip, Årsta, Sweden). Simultaneously, both the vehicle and the ICI group were given daily subcutaneous (s.c.) injections of 17β-estradiol-3-benzoate (E2) (Sigma, St Louis, MO, USA) (0.7 μg/day), 4-estren-3α, 17β-diol (estren) (Steraloids Inc., Newport, RI, USA) (75 μg/day) or control Miglyol 812 oil for 18–21 days. Each group consisted of 6–10 mice.

#### Experiment 2

Male and female double heterozygous (ERα^+/-^β^+/-^) mice on a mixed C57B1/6J/129 background, were mated resulting in WT (ERα^+/+^β^+/+^), ERKO (ERα^-/-^β^+/+^), BERKO (ERα^+/+^β^-/-^) and DERKO (ERα^-/-^β^-/-^) offspring[24,25]. Genotyping of tail DNA was performed as previously described[26,27]. 11-month-old female WT and DERKO mice were ovariectomized under Ketalar^®^/Domitor^® ^anaesthesia. Mice were given daily s.c. injections of E2 (0.7 μg/day), 5α-dihydrotestosterone (DHT) (Sigma) (120 μg/day) or estren (75 μg/day) during four weeks. Control mice received vehicle olive oil (Apoteksbolaget, Sweden). Each group consisted of 6–8 mice.

#### Experiment 3

9-month-old male WT and DERKO mice were orchidectomized under Ketalar^®^/Domitor^® ^anaesthesia. Mice were treated for 4 weeks with E2 (0.05 μg/day) or DHT (45 μg/day), administered by s.c. silastic implants (Silclear Tubing, Degania Silicone, Jordan Valley, Israel) in the cervical region[18]. Vehicle animals received empty implants. Each group consisted of 4–8 mice.

#### Experiment 4

Untreated 18-month-old female WT, ERKO, BERKO, and DERKO mice were used in the experiment. Each group consisted of 7–8 mice.

### Tissue collection and single-cell preparation

Mice were anaesthetised by Ketalar^®^/Domitor^® ^, bled and killed by cervical dislocation. SMG were dissected, weighed and fixed in paraformaldehyd. Thymi were removed, weighed and single-cell suspensions were prepared by mashing the organs through a 70 μm cellstrainer (BD, Franklin Lakes, NJ, USA). Bone marrow cells were harvested from femur and tibia by flushing with 2 ml Phosphate Buffered Saline (PBS). Cells were kept in a 50/50 mixture of complete medium (Iscoves-medium enriched with 50 μg/ml Gentamicin (Sigma), 4 mM L-glutamine (Sigma), 50 μM Mercaptoethanol (Sigma) and 10 % Fetal Calf Serum (FCS) (Biological Ind., Beit Haemek, Israel)) and PBS until use. Thymus and bone marrow cells were centifuged at 515 × g for 5 min. Pelleted bone marrow cells were re-suspended in Tris-buffered 0.83% NH_4_Cl solution (pH 7.29) for 5 minutes to lyse erythrocytes. After washing in PBS the total number of thymus and bone marrow cells was calculated using an automated cell counter (Sysmex, Kobe, Japan). Cells were re-suspended in complete medium before use.

### Flow cytometry

Bone marrow cells were subjected to Fluorescence Activated Cell Sorter (FACS) analysis. Cells were stained with fluorescein isothiocyanate (FITC) labelled antibodies to CD45R/B220 (clone RA3-6B2, BD PharMingen) and allophycocyanin (APC) conjugated antibodies to CD 19 (clone 1D3, BD PharMingen). Row cytometry was performed on a FACSCalibur and analyzed using Paint-A-Gate software (BD). The FACS data are presented as percentage positively stained bone marrow cells of all nucleated cells.

### Histological examination

Histological examination of SMG was performed in a light microscope (×100) after routine 4% paraformaldehyde fixation, dehydration, paraffin embedding and preparation of 4 μm thick tissue sections, followed by staining with hematoxylin and eosin[28]. The degree of the androgen phenotype in the SMG sections were scored from 0 to 3, where 0 represents no androgen phenotype and 3 is the highest score for androgen phenotype. Representative pictures for the different scorings (0–3) are presented in fig. 3.

### Statistical analysis

All values were first tested for normal distribution using the Shapiro-Wilk test. One-way ANOVA followed by Fisher's test were then used to compare data from mice in different treatment groups (experiment 1–3), or from mice of different genotypes (experiment 4). Results are presented as mean ± standard deviation. Two-sided testes were used and *P *< 0.05 was considered statistically significant. The programs Prism 4.0 and Statview 5.0.1 were used for statistical calculations.

## List of abbreviations

AR: androgen receptor

DERKO: double estrogen receptor knock-out

DHT: 5α-dihydrotestosterone

E2: 17β-estradiol

ER: estrogen receptor

ERα: estrogen receptor α

ERβ: estrogen receptor β

estren: 4-estren-3α,17β-diol

GCT: granular convoluted tubules

ORX: orchidectomized

OVX: ovariectomized

s.c.: subcutaneously

SMG: submandibular gland

WT: wild type

## Authors' contributions

**UI **participated in the planning, termination and execution of experiments, analysed and interpreted data, drafted the manuscript.

**BH **participated in the termination of experiments, analysed and interpreted the submandibular gland sections, critically reviewed the manuscript.

**MCE **participated in the termination and execution of experiments and analysed interpreted data, critically reviewed the manuscript.

**CJ **participated in the termination and execution of experiments, critically reviewed the manuscript.

**SMS **participated in the planning, termination and execution of experiments, critically reviewed the manuscript.

**ML **participated in the planning, termination and execution of experiments, critically reviewed the manuscript.

**JÅG **provided the estrogen receptor knock-out mice and participated in the design of the studies, critically reviewed the manuscript.

**CO **conceived of the studies, and participated in their design and coordination, critically reviewed the manuscript.

**HC **conceived of the studies, and participated in their design, coordination and execution, helped to draft the manuscript.
